# Responses of Antioxidant Defense and Immune Gene Expression in Early Life Stages of Large Yellow Croaker (*Pseudosciaena crocea*) Under Methyl Mercury Exposure

**DOI:** 10.3389/fphys.2018.01436

**Published:** 2018-10-10

**Authors:** Fangzhu Wu, Wei Huang, Qiang Liu, Xiaoqun Xu, Jiangning Zeng, Liang Cao, Ji Hu, Xudan Xu, Yuexin Gao, Shenghua Jia

**Affiliations:** ^1^Key Laboratory of Marine Ecosystem and Biogeochemistry, Second Institute of Oceanography, State Oceanic Administration, Hangzhou, China; ^2^Ocean College, Zhejiang University, Hangzhou, China; ^3^Institute of Oceanology, Chinese Academy of Sciences, Qingdao, China; ^4^Zhejiang Surveying Institute of Estuary and Coast, Hangzhou, China

**Keywords:** large yellow croaker, juvenile, MeHg exposure, antioxidant defense system, mRNA expression

## Abstract

Early life stages of marine organisms are the most sensitive stages to environment stressors including pollutants. In order to understand the toxicological effects induced by MeHg exposure on juveniles of large yellow croaker (*Pseudosciaena crocea*), a toxicity test was performed wherein fish were exposed to sub-lethal concentrations of MeHg under laboratory conditions (18 ± 1°C; 26 ± 1 in salinity). After 30 days of 0–4.0 μg L^-1^ MeHg exposure, SOD activity was significantly decreased in the 0.25, 1.0, and 4.0 μg L^-1^ treatments; while CAT activity was significantly increased in the 4.0 μg L^-1^ treatments; GSH level, GPx activity were significantly elevated in the 4.0 μg L^-1^ treatments, respectively. Meanwhile, malondialdehyde content was also significantly increased in the 1.0 and 4.0 μg L^-1^ treatments with respect to the control. Acetylcholinesterase activity was significantly decreased by 18.3, 25.2, and 21.7% in the 0.25, 1.0, and 4.0 μg L^-1^ treatments, respectively. The expression of *TCTP*, *GST3*, *Hsp70*, *Hsp27* mRNA were all up-regulated in juveniles with a dose-dependent manner exposed to MeHg. These results suggest that large yellow croaker juveniles have the potential to regulate the levels of antioxidant enzymes and initiate immune response in order to protect fish to some extent from oxidative stress induced by MeHg.

## Introduction

Mercury (Hg) is one of the most toxic metals in the global environment which widely exists in lithosphere, hydrosphere, atmosphere and biosphere, its main anthropogenic sources are urban and industrial emissions, agricultural materials, mining, and combustion (Zhang and Wong, 2007). In marine ecosystems, Hg exists in a variety of forms such as inorganic salts, metallic elements, and organic compounds (Lee et al., 2017b). The most toxic form of Hg is methyl mercury (MeHg) which is mainly synthetized through microbial processes and biochemical reactions in aquatic ecosystems (Fitzgerald and Mason, 1997). MeHg is the most highly absorbable form of Hg and it is primarily bioaccumulated in aquatic life, the percentage of MeHg is about 72–100% to total Hg in fish (Arleny et al., 2007; Zhang and Wong, 2007). Bioaccumulation of MeHg in fish rests with the trophic level as well as its age, length, or weight (Zhang and Wong, 2007), the concentration of MeHg in top predator fish could be three orders of that in ambient waters (Morel et al., 1998). As the ultimate predator at the top of food chain, human beings also have high risk exposed to Hg, primarily by eating fish contain MeHg (Squadrone et al., 2015).

MeHg is known as its neurotoxicity which can damage the central nervous system (CNS) of organisms (Kaur et al., 2006). It can penetrate the blood–brain barrier easily with L-type amino acid transporters such as the MeHg -L-cysteine complex (Aschner, 1989). Moreover, MeHg has been proven to injure immune system, alter enzymes system, induce DNA damage as well (Cambier et al., 2009; Squadrone et al., 2015). For fish, previous studies indicated that MeHg could affect the gonadal development, reproduction, feeding behavior, and induce oxidative damage (Mozhdeganloo et al., 2015). For example, delayed mortality syndrome appeared in zebrafish (*Danio rerio*) after embryonic exposure below 20 μg L^-1^ MeHg (Samson et al., 2001). Besides, mitochondrial bioenergetics can be disturbed in skeletal muscle of adult zebrafish (*Danio rerio*) by low concentration of MeHg exposure (Cambier et al., 2009). Early life stages (ELSs) have been proved to be particularly sensitive to environment stressors including pollutants (Devlin and Mottet, 1992). Fish exposed to low concentrations of pollutants during their ELS may induce injury on biology and behavior to their later developmental stages (Devlin, 2006). Thus, the toxicity evaluation derived from these ELS experiments could strongly indicate the range of potential biological effects of toxicant action (Devlin, 2006).

In China, the concentrations of total Hg (THg) and MeHg in the surface seawater of northern South China Sea ranged from 0.0008–0.0023 μg L^-1^ and 0.00005–0.00022 μg L^-1^ (Fu et al., 2010). While in the Bohai Sea and East China Sea, THg mercury ranged from 0.002–0.15 μg L^-1^ and 0.012–0.071 μg L^-1^, respectively (Huang et al., 2010b). Large yellow croaker (*Pseudosciaena crocea*) are middle carnivorous fish which mainly distributed in the coastal waters of southeast China (Xu and Liu, 2007; Xia et al., 2013), which used to be the most important commercial fish in China, however, their wild population and resources decreased dramatically since the 1980s (Xu and Liu, 2007). Meanwhile, the pollutants including metals in the waters and sediments of their spawning and nursery grounds in the East China Sea had been overloaded (Wang et al., 2005). Compared with lower carnivorous fish and omnivorous fish, large yellow croaker accumulated higher levels of THg and MeHg due to their higher trophic position and larger size. It has been reported that the concentrations of THg and MeHg accumulated in large yellow croaker from coasts of East China are 34.4 and 24.9 ng g^-1^, respectively (Xia et al., 2013). Therefore, biological damages caused by contaminants on reproduction, development, and survival of large yellow croaker have been considered as potential hazards for their wild population deterioration (Wang et al., 2005). However, studies on the toxicity of pollutants to the ELS of large yellow croaker are limited with that of heavy metal exposure being particularly lacking.

In order to better understand the toxicological effects of MeHg to large yellow croaker, a toxicity test was performed wherein juveniles were exposed to sub-lethal concentrations of MeHg for 30 days. The specific objectives of the present study were as follows: (1) to elucidate how antioxidants (SOD, CAT, GSH, GPx) responded to MeHg accumulation to cope with oxidative stress; (2) to determine how lipid peroxidation and acetylcholinesterase (AChE) responded to MeHg exposure; (3) to evaluate the mRNA expression of genes related to immune response (*TCTP*, *GST3*, *Hsp70*, *Hsp27* transcripts) by MeHg exposure.

## Materials and Methods

### Experimental Organisms

This study was carried out in accordance with the recommendations of Second Institute of Oceanograpy, State Oceanic Administration, China. Large yellow croaker juveniles were obtained from Institute of Marine and Fisheries Research of Ningbo, China. The fish were acclimatized in a flow-through pond with filtered seawater (hardness, 6124.6 ± 272.3 mg L^-1^ as CaCO_3_; pH, 8.1 ± 0.1; salinity, 28 ± 1; dissolved oxygen, 7.5 ± 0.2 mg L^-1^; MeHg, 0.00006 ± 0.00003 μg L^-1^) for 2 weeks before they were used in experiments. During acclimation, the fish were fed artificial foods (Tianma Company, Fujian, China) twice a day with a light regime of 14L:10D. A thermostat-controlled water bath system was used to maintain the water temperature at 18 ± 1°C.

### Experimental Procedure

About 1,000 healthy juvenile fish with similar size were selected randomly and transferred into each of the 300 L experimental tanks, which were placed randomly in the laboratory with an constant temperature of 18 ± 1°C. Fish size (2.0 ± 0.2 cm in total length, L_T_; 0.13 ± 0.04 g in body weight, W_B_) did not significantly differ between replicates or concentrations (ANOVA, *p* > 0.05 in all cases). After a 24 h acclimation period in the experimental tanks without feeding, the fish were exposed to either a blank control (0 μg L^-1^) solution or a MeHg solution of 0.25, 1.0, or 4.0 μg L^-1^ MeHg. Four replicates were performed for each concentration. Each experimental tank was filled with 200 L of filtered seawater. The fish rearing conditions were identical to those for the acclimation described above. The analytical-reagent methyl mercury chloride (CH_3_ClHg, purity over 99.5%; CAS No: 115-09-3; Sigma-Aldrich Chemical, Co., United States) was used as the test chemical. A stock solution (1.0 g L^-1^) was prepared by dissolving CH_3_ClHg in deionized water, from which appropriate aliquots were diluted in filtered seawater to obtain the designated concentration in each experimental tank. Oxygen was gently provided by a continuous air-bubbling system. The fish were fed artificial foods to satiety at 9:00, excessive food, dead juveniles were removed and the solution in the experimental tanks was renewed daily with freshly prepared solution of the same MeHg concentration at 15:00 during the test. The test was terminated 30 days after exposure began. The test solutions were sampled from each experimental beaker at the beginning, the intermediate and the end of the tests, respectively. Test solutions were measured using gas chromatography–mass spectrometry (GC-MS) (Agilent 6890/5973; United States). The measured concentrations fell within the range of 80–120% of the nominal concentrations.

At the end of test, 100 fish were randomly sampled from each experimental tank. They were freshly weighed to the nearest 0.1 g (W_B_) and measured to the nearest 0.1 cm (L_T_) for growth determination. Fish size (4.06 ± 0.61 cm in total length, L_T_; 0.47 ± 0.17 g in body weight, W_B_) did not significantly differ between replicates or concentrations (ANOVA, *p* > 0.05 in all cases). After that, the fish were frozen immediately in freezing tube in the -80°C refrigerator (Thermo Scientific 902-ULTS). The tissues were then immediately stored in acid-rinsed vials in liquid nitrogen awaiting the determination of antioxidant and the expression of mRNA.

### Antioxidant Enzymes and Lipid Peroxides Determination

Samples used for enzymes determination were thoroughly washed with ice-cold physiological salt solution (0.9% NaCl), surface dried with absorbent paper and accurate weighted. Then the samples and 10 mM ice-cold Tris-HCl buffer solution (pH 7.4) at the ratio of 1: 9 by weight to volume were transferred into a glass tube and homogenized by a homogenizer (IKA, Germany) with ice bath. The tissue fluid was centrifuged for 10 min at 12000 rpm at 4°C (Eppendorf 5804R, Hamburg, Germany). The supernatants were collected for further analysis.

#### Superoxide Dismutase (SOD) Activity

The SOD activity was determined by method of pyrogallol auto-oxidation (Marklund and Marklund, 1974). Add 8.7 mL of 50 mM phosphoric acid buffer (pH 8.2) and 0.25 mL of 20 mM pyrogallol to 50 μL supernatant at 25°C and the determination wavelength is 325 nm. One SOD activity unit was defined as the amount of enzyme exhibiting 50% inhibition of the auto-oxidation rate of 0.1 mM pyrogallol per minute in 1 mL solution at 25°C. Results were expressed as U mg^-1^ Pr.

#### Catalase (CAT) Activity

Activity of CAT was measured with reference to the method of Beers and Sizer (1952). Add 1.9 mL of 50 mM phosphoric acid buffer (pH 7.0) and 1.0 mL of 5 mM H_2_O_2_ to 100 μL supernatant and the determination wavelength is 240 nm. One CAT activity unit was defined as the amount of enzyme that catalyzed the degradation of 1 μmol H_2_O_2_ per minute and results were expressed as U mg^-1^ Pr.

#### Glutathione (GSH) Content

Glutathione content was measured with reference to the method of Hissin and Hilf (1976). Add 4 mL phosphoric acid-EDTA buffer (pH 8.0) to 1 mL supernatant and mixed thoroughly. Take 100 μL diluted supernatant added with 1.8 mL phosphoric acid-EDTA buffer (pH 8.0) and 100 μL orthophthalaldehyde (1 mg mL^-1^). After thorough mixing and incubation at room temperature for 15 min and fluorescence was determined at 420 nm. Results were expressed as μg mg^-1^ Pr.

#### Glutathione Peroxidase (GPx) Activity

Activity of GPx was measured by method of Rotruck et al. (1973). Added 200 μL of 400 mM Tris buffer (pH 7.0), 100 μL of 10 mM sodium azide (NaN_3_), 200 μL of 1 mM GSH and 100 μL of 0.2 mM H_2_O_2_ to 200 μL supernatant and mixed thoroughly at 37°C water bath for 10 min; then added 0.4 mL of 10% TCA to end the reaction and collected the supernatant after centrifuging for 15 min at 3000 rpm. Added 0.5 mL Ellam solution (19.8 mg DTNB dissolved in 100 mL of 0.1% sodium nitrate) (NaNO_3_) and 3.0 mL phosphoric acid-EDTA buffer (pH 8.0) to 1.0 mL collected supernatant then recorded the absorbance at 412 nm. One GPx activity unit was defined as the amount of enzyme required to deplete 1 μM GSH in per mg protein per minute and results were expressed as U mg^-1^ Pr.

#### Malondialdehyde (MDA) Content

Malondialdehyde (MDA) content was measured by method of TBA (2-thiobarbituric acid) colorimetry improved by Luo et al. (2006). Add 200 μL supernatant, 0.2 mL of 8.1% sodium laurylsulfonate, 1.5 mL of 20% acetic acid buffer (pH 3.5), 1.5 mL of 1% TBA and 1 mL distilled water in order and mixed thoroughly at 95°C water bath for 80 min then cooled and centrifuged for 15 min at 3000 rpm. Collected the supernatant and recorded the absorbance at 532 nm. MDA content is expressed as the amount of TBARS contained in per mg protein and results were expressed as nmol mg^-1^ Pr.

Total protein content in tissues was estimated by method of Bradford (1976). The absorbance was recorded at 595 nm with bovine serum protein (BSA) as the standard protein. All measurements above were performed with UNICO ultraviolet-visible spectrophotometer (UNICO WFZ UV-2802PC/PCS; Shanghai, China).

### Acetylcholinesterase (AChE) Activity

Activity of AChE was measured following the methodology first described by Ellman et al. (1961) and adapted to 96-well microplates. 400 μL supernatant was added to a cuvette containing 2.6 mL of phosphate buffer (pH 8.0, 0.1 M). Samples absorbance was read at 412 nm, every minute during 10 min. AChE activity was measured considering that one unit of enzyme is responsible for the formation of 1.0 nmol of thiocholine per minute. Results were expressed as U mg^-1^ Pr.

### *TCTP, GST3, Hsp70, Hsp27* mRNA Expression

Total RNA was extracted from *Pseudosciaena crocea* using the Trizol reagent (Invitrogen, United States) according to the manufacturer’s directions. Total RNA was incubated with RQ1 RNase-free DNase (Promega, United States) to remove the contaminating genomic DNA. cDNA was transcribed from 1 mg of total RNA using SuperScript III kit (Invitrogen, United States) according to the manufacturer’s guide. *TCTP*, *GST3*, *Hsp70*, *Hsp27* mRNA expression levels were determined using the ABI PRISM 7000 Fluorescent Quantitative PCR System (Applied Biosystems, United States) with the *β-actin* gene as a reference gene. Quantitative real-time polymerase chain reaction (qPCR) was performed in a total volume of 50 μL containing 25 μL of SYBR Green PCR Master Mix, 1 μL of a mixture of forward and reverse primers (10 μmol/L), 4 μL of nuclease free water and 20 μL of the diluted cDNA templates. The qPCR cycles were as follows: 65°C for 2 min, 95°C for 10 min followed by 40 cycles of 95°C for 15 s and 65°C for 1 min. All qPCR reactions were performed in triplicate. At the end of each qPCR reaction, dissociation curve analysis of the amplification products was carried out to confirm that only one PCR product was amplified. The 2^-ΔΔCT^ method was employed to calculate the relative mRNA expression level of the *TCTP*, *GST3*, *Hsp70*, *Hsp27* mRNAs. The *β-actin* gene was constitutively expressed at constant level before and after MeHg treatment, indicating the stability of the reference gene. The primers were listed in **Table 1**.

**Table 1:** Primers used in the experiment.

Primer name	Primer sequence (5′–3′)	Purpose
TCTP-F	CAAAGCGCCAACATGATCAT	RT-PCR and quantitative
TCTP-R	CACCTGGTATGACTTCTTGTTGTA	Real-time PCR
GST3-F	CCCTCTGAACTAATACAAAATCA	
GST3-R	GCTCTAACGCCATCGTTGTCTGC	
Hsp27-F	ACACTCCTCTACAGCCATGA	
Hsp27-R	CAGCTCCTCGGGTGAAAAGTGGT	
Hsp70-F	GAGACTGCGGGTGGAGTTATG	
Hsp70-R	TGGCTCTCTCACCCTCATACAC	
β-Actin-F	TTATGAAGGCTATGCCCTGCC	
β-Actin-R	TGAAGGAGTAGCCACGCTCTGT	


### Data Treatment and Statistical Analyses

The results of the experiment were expressed as Means ± standard deviation (means ± SD), Kolmogorov–Smirnov test and Levene test were used to test the hypothesis of normality and homogeneity respectively. If the data satisfied both normality and homogeneity, one-way ANOVA and Dunnett’s multiple comparison method were used to analyze the significant differences between groups. If either of the two assumptions was not satisfied, the data would be converted logarithmically and then the ANOVA and multiple comparisons would be performed. All statistical analysis was carried out with SPSS software (SPSS 15.0, Chicago, IL, United States) and significance level of *p* < 0.05 was used for all tests. Software SigmaPlot 12.5 was used to do figure processing.

## Results

### Effects on Antioxidants in the Tissues of *Pseudosciaena crocea*

**FIGURE 1:**
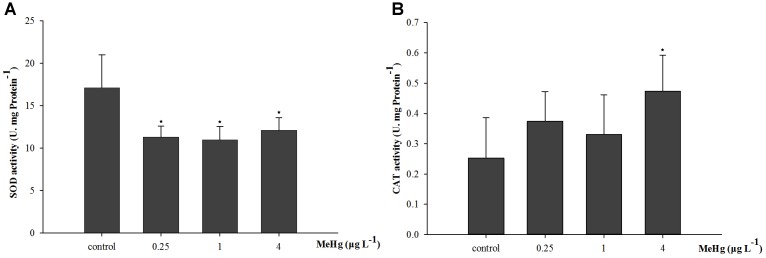
Changes of **(A)** SOD activity, and **(B)** CAT activity in *Pseudosciaena crocea* under 30 days exposure to MeHg (U mg^-1^ Pr, value represent means ± SD), *n* = 4, asterisks represent statistically significant differences compared with the control (^∗^*p* < 0.05).

After exposed to MeHg for 30 days, the SOD activity of large yellow croaker juveniles was significantly inhibited (ANOVA, *p* < 0.05), it was 33.9, 35.8, and 29.2% lower in the 0.25, 1.0, and 4.0 μg L^-1^ groups than in the control, respectively (Dunnett’s test, *p* < 0.05; **Figure 1A**). The CAT activity of juvenile fish was also significantly affected by MeHg exposure, it was 87.7% higher in the 4.0 μg L^-1^ MeHg treatment group with respect to the control (ANOVA Dunnett’s test, *p* < 0.05; **Figure 1B**).

**FIGURE 2:**
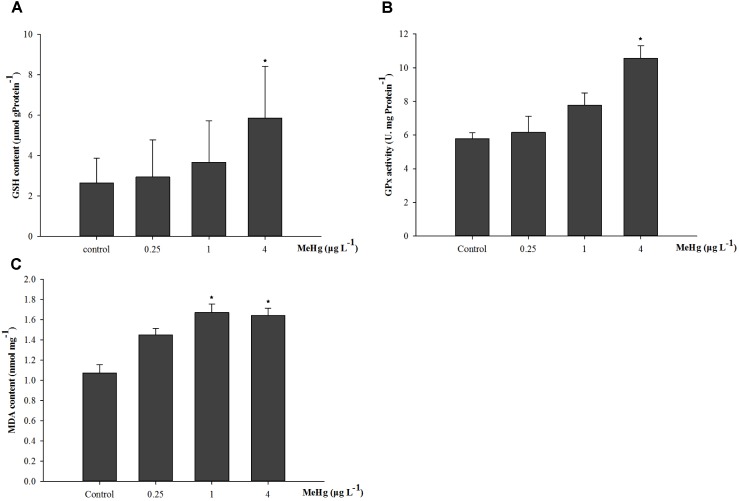
Changes of **(A)** GSH content, **(B)** GPx activity, and **(C)** MDA content in *Pseudosciaena crocea* under 30 days exposure to MeHg (μg mg^-1^ Pr, U mg^-1^ Pr, nmol mg^-1^, value represent means ± SD), *n* = 4, asterisks represent statistically significant differences compared with the control (^∗^*p* < 0.05).

The GSH content of juvenile was significantly induced by MeHg exposure (ANOVA, *p* < 0.05), and results showed that GSH content in 4.0 μg L^-1^ group increased by 121.5% compared with that in the control group (ANOVA, Dunnett ’s test, *p* < 0.05; **Figure 2A**). GPx activity was also significantly induced (ANOVA, *p* < 0.05), GPx activity in the 1.0 and 4.0 μg L^-1^ groups increased by 34.5 and 82.7% than control after 30 days MeHg exposure (Dunnett’s test, *p* < 0.05; **Figure 2B**). As for the MDA content, it was 56.3 and 53.5% higher in the 1.0 and 4.0 treatments than that in the control group, respectively (ANOVA Dunnett’s test, *p* < 0.05; **Figure 2C**).

**FIGURE 3:**
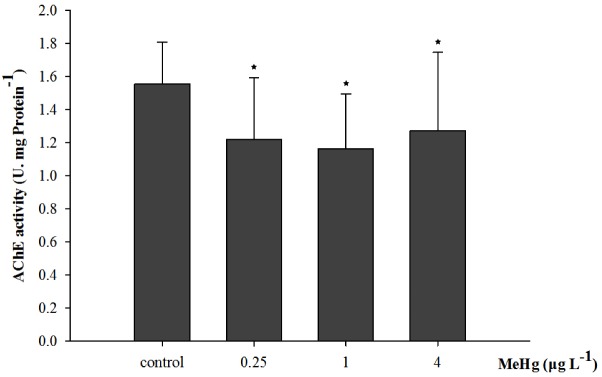
Changes of acetylcholinesterase (AChE) activity in *Pseudosciaena crocea* under 30 days exposure to MeHg (U mg^-1^ Pr, value represent means ± SD), *n* = 4, asterisks represent statistically significant differences compared with the control (^∗^*p* < 0.05).

Acetylcholinesterase activity was significantly affected by 30 days of MeHg exposure (ANOVA, *p* < 0.05). It decreased by 18.3, 25.2, and 21.7% in the 0.25, 1.0, and 4.0 μg L^-1^ group respectively comparing with the control (Dunnett’s test, *p* < 0.05; **Figure 3**).

### Expression Profiles of *TCTP*, *GST3*, *Hsp70*, *Hsp27* in *Pseudosciaena crocea* After Exposed to MeHg

**FIGURE 4:**
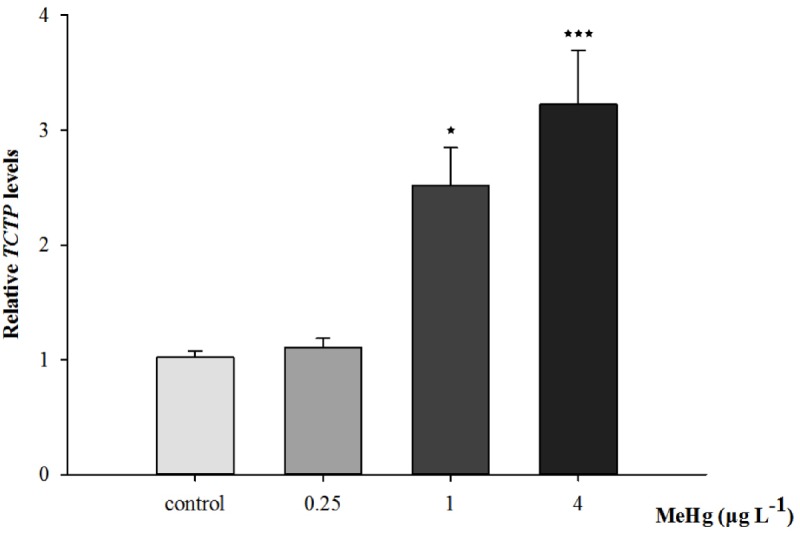
Changes of TCTP mRNA expression level in *Larimichthys crocea* under 30 days exposure to MeHg (value represent means ± SD), *n* = 3 (^∗^*p* < 0.05; ^∗∗∗^*p* < 0.001).

**FIGURE 5:**
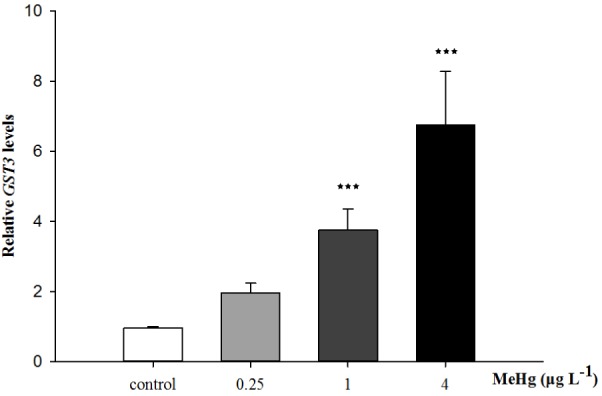
Changes of GST3 mRNA expression level in *Larimichthys crocea* under 30 days exposure to MeHg (value represent means ± SD), *n* = 3 (^∗∗∗^*p* < 0.001).

**FIGURE 6:**
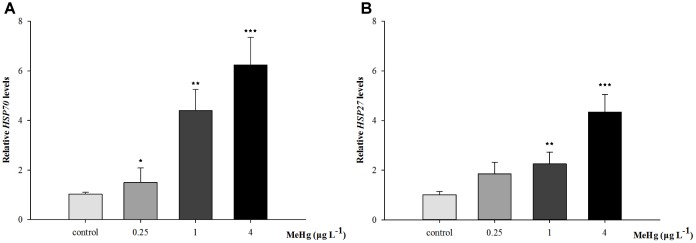
**(A)** *Hsp70*, **(B)** *Hsp27* mRNA expression level in (*Larimichthys crocea*) after expose to various doses of MeHg. The results are shown as value represent means ± SD (*n* = 3), ^∗^*p* < 0.05; ^∗∗^*p* < 0.01; ^∗∗∗^*p* < 0.001.

Quantitative real-time polymerase chain reaction was performed to measure the expression of *TCTP*, *GST3*, *Hsp70*, *Hsp27*, transcripts in muscle with *β-actin* employed as the normalization gene. The mRNA expression levels of *TCTP*, *GST3*, *Hsp70*, *Hsp27* were all significantly up-regulated in *Pseudosciaena crocea* in a dose-dependent manner after treatment of MeHg (**Figures 4, 5, 6A,B**).

## Discussion

### Antioxidant Defense

Antioxidants as well as free radical scavengers levels have been proved to be closely related with various stressors including metals (Aliko et al., 2018). The first reaction to ambient pressure is oxidative stress and it usually occurs when the ROS level exceeds the scavenging activities of antioxidant molecules (Maharajan et al., 2018). Pollutants including MeHg were thought to be effective inducer for ROS formation in organisms (do Nascimento et al., 2008). Previous studies indicated that this event closely related to cell energy metabolism dysfunction and electron transport chain disruption (do Nascimento et al., 2008). Excessive ROS production could cause the decrease of mitochondrial membrane, which may lead to the oxidation of polyunsaturated fatty acids of membrane and produce a large amount of lipid peroxidation (Yin et al., 2007).

SOD is the enzyme that catalyze $O_{2}^{-}$ and H^+^ to less toxic H_2_O_2_ and O_2_ while CAT converts H_2_O_2_ into H_2_O (Maharajan et al., 2018), thus to prevent oxidative stress and maintain cell homeostasis (Mansour and Mossa, 2009). Therefore, the SOD-CAT system provide the first line of defense against ROS damage in organisms (Liu et al., 2015). Previous research suggested that the activity of SOD could be activated to dispel the increased ROS in cells (Zhao et al., 2013). In the present study, SOD activity was inhibited by MeHg exposure, this phenomenon was probably due to the antioxidant failed to dispel the excessive ROS induced by MeHg. Similar results was also reported in liver and brain of seabass (*Dicentrarchus labrax*) by MeHg exposure (Maulvault et al., 2017). MeHg could induce a reduction of activity in enzymes related to mitochondrial energy metabolism including SOD, which could be explained by MeHg may impair the function of mitochondria when the organelle exhibits a membrane permeability transition state (do Nascimento et al., 2008). However, opposite results were found in liver and kidney of Atlantic salmon (*Salmo salar*) parr and zebrafish (*Danio rerio*) which indicated an adaptive reaction to neutralize oxidative stress was induced by MeHg exposure (Berntssen et al., 2003; Strungaru et al., 2018). Moreover, SOD activity may change with the exposure concentration, for example, increased SOD activity was found in the brain of Atlantic salmon (*Salmo salar*) parr by 5 mg MeHg/kg exposure, but with the concentration increased to 10 mg MeHg/kg decreased activity occurred (Berntssen et al., 2003). This result may explained by the brain is a relatively susceptible organ to oxidative injury compared with liver and kidney, and the redox defense system in the brain is more easier to break-down after higher concentration of MeHg exposures (Berntssen et al., 2003).

The induction of CAT activity observed in the present study may be related to oxidative stress leading to the continuous generation of H_2_O_2_ by $O_{2}^{-}$, and activated CAT activity could protect the organism by scavenging H_2_O_2_. This was consistent with liver and muscle of juveniles seabass (*Dicentrarchus labrax*) exposed to MeHg (Maulvault et al., 2017). Similarly, the gene expression of *cat* was also found to be up-regulated by MeHg exposure in glass eel (*Anguilla anguilla*) (Claveau et al., 2015). On the contrary, inhibited CAT activity was found in zebrafish (*Danio rerio*) larvae by mercury chloride exposure (Cruz et al., 2013). Such various results in previous studies indicated that activity of SOD and CAT responded differently to mercury-related exposure in different species and different life stages, some species showing increased activity while others exhibiting inhibited activity or no responses (Cao et al., 2010). In addition, various responses of antioxidant enzymes are also related with concentration of MeHg, exposure time and environmental factors such as temperature, etc. (Huang et al., 2010a).

Glutathione is thought to be one of the most important non-protein thiols in organisms’ cell and tissue (Hoguet and Key, 2007). It has the potential to prevent cellular components damage caused by free radicals (Pompella and Corti, 2003). GSH-redox system is known to be a key antioxidant defense in protecting cells from oxidative damage as well as detoxification (Lee et al., 2017b). Several studies reported decreased GSH level by MeHg exposure, for example, decreased GSH was observed in liver of rainbow trout (*Oncorhynchus mykiss*) and copepod (*Tigriopus japonicus*) by MeHg exposure, which could be explained by the formation of MeHg-GSH complex by interaction of MeHg with GSH through its SH group or enhanced oxidation of this thiol (Mozhdeganloo et al., 2015; Lee et al., 2017a). In addition, changes of expression levels of genes related to GSH metabolism in brain of female zebrafish (*Danio rerio*) reflects MeHg exposure may lead to the metabolic dysfunction of GSH (Richter et al., 2011). However, in the present study, GSH content was increased after 30 days of MeHg exposure. Similar results was also found in liver of matrinxa (*Brycon amazonicus*) exposed to mercury chloride which could be explained by the fish gut could protect themself from oxidative damage through enhance uptake of amino acid substrates and the biosynthetic enzymes related to GSH synthesis when encounter mercury exposure (Monteiro et al., 2010). In summary, the alteration of GSH content and metabolism are of vital importance in protecting organisms against oxidative stress induced by MeHg.

GPx could convert hydroperoxides into hydroxyl compounds with GSH as substrate and its main detoxification function is to terminate the propagation of the radical chain, thus protecting membranes from oxidative damage (Maharajan et al., 2018). As an effective antioxidant defense system of oxidative stress induced by MeHg, the increased GPx activity is associated with a compensatory response of SOD activities (Lee et al., 2017a). Therefore, significantly induced GPx activity in this study might indicate these molecules were participated in the detoxification of H_2_O_2_ produced in juveniles. This result is consist with copepod (*Tigriopus japonicus*) exposed to MeHg (Lee et al., 2017a). On the other hand, in the rotifer (*Brachionus koreanus*), the GPx activity was inhibited when exposed to low concentration of MeHg (1 and 10 ng/L), but increased GPx activity was observed with the concentration increased (100 ng/L) (Lee et al., 2017b).

Malondialdehyde is the product of lipid peroxidation formed by lipoperoxidation reaction of oxygen free radicals, phospholipids in biofilm and lipoperoxidation of macro molecules such as side chains of polyunsaturated fatty acids and nucleic acids associated with enzymes and membrane receptors (Uner et al., 2001). Expose to exogenous pollutants could induce a large number of MDA and its content could directly reflect the level of oxidative stress, so it is an important index to evaluate the degree of oxidative damage in cells (Mozhdeganloo et al., 2015). In the present study, MDA content of juveniles was significantly induced by MeHg exposure. Increased MDA were also reported in zebrafish (*Danio rerio*) and rainbow trout (*Oncorhynchus mykiss*) by MeHg exposure (Mozhdeganloo et al., 2015; Strungaru et al., 2018). The results suggested that although the antioxidant synthesized of large yellow croaker was induced to some extent by MeHg exposure, but it was not sufficient to eliminate the large amount of ROS produced. The degree of lipid peroxidation was aggravated which might exceed the ability of anti-oxidation and defense in large yellow croaker, aggravate the degree of oxidative damage in tissues and finally caused a series of metabolic and functional disorders, which had a toxic effect on cells.

### AChE Activity

The toxicity of MeHg mainly targets at CNS, it alters the biochemical and ultrastructure of both astrocytes and neurons (Lee et al., 2017b). Previous researches showed that the developing CNS is more sensitive to several contaminants than the adult CNS (Farina et al., 2011). MeHg affects the cerebral cortex, visual, auditory, somatic sensory, and motor cortex, as well as the hippocampus and the granule layer of the cerebellum in the adult brain, thus causing a remarkable loss of neurons in these brain regions (Carta et al., 2003). However, due to the high sensitivity of the immature CNS in the developing brain, a widespread neuronal loss is more easier to occur (do Nascimento et al., 2008). As an important enzyme in nervous system, AChE could hydrolyze the neurotransmitter acetylcholine (ACh), and AChE activity is widely used in assessment of neurotoxic stress induced by pollutants (Hoguet and Key, 2007). In the present study, AChE activity was inhibited by MeHg exposure, similar results was also observed in juveniles of seabass (*Dicentrarchus labrax*), since MeHg might be strongly combined with the AChE receptor, thus blocking the electric signals between neurons and target cells (Maulvault et al., 2017). Inhibited AChE activity in the present study may indicate the neurotoxicity caused by MeHg. However, further research about juveniles behavior, histology and biochemical analysis of brain are still needed to clarify the neurotoxicity induced by MeHg exposure.

### Gene Expressions Related to Immune Response

Parental compounds of environmental contaminants, their metabolites or indirectly generation of ROS may result in DNA damage in organisms (Oliveira et al., 2009). In order to investigate the immune response in detail, the mRNA expression of essential proteins related to antioxidant enzymes were investigated. *TCTP* is a highly conserved multifunctional protein which is usually highly expressed in tumor cells with activities ranging from cytoskeletal to transcription regulation in organisms (Liu et al., 2018). It also regulates the innate immune response, especially the inflammatory to neutralize infectious substances and initiate repair to the injured tissue (Jia et al., 2017). Accumulating evidence indicated that *TCTP* also participated in the anti-stress responses in invertebrate (Jia et al., 2017). For instance, in nematode (*Brugia malayi*), *BmTCTP* could serve as an anti-apoptotic protein and protect cells from oxidative damage (Gnanasekar and Ramaswamy, 2007). *TCTP* was also an anti-metal stress factor, for instance *MgTCTP* of the mussel (*Mytilus galloprovincialis*) could participated in the regulation of cadmium stress (You et al., 2013). In the present study, *TCTP* expression level was significantly up-regulated in a dose-dependent manner after MeHg treatment which indicate that adaptive immune response to stress may be caused by MeHg. This result is consistent with crustacean (*Eriocheir sinensis*) by copper exposure, since heavy metal ions may be accumulated in tissues, and the synthesis of *TCTP* increases correspondingly with the increase of ions (Wang et al., 2011). In the future, the specific molecular mechanisms underlying the response of *TCTP* to stressors such as heavy metal require to be revealed, especially whether this gene can be applied as a molecular biomaker in ecotoxicological studies.

Glutathione *S*-transferase (GSTs), a widely expression protein that can be found in various tissues, possesses detoxification and antioxidation abilities and plays an important role in immune and biotransformation (Leiers et al., 2003). One of the major functions of GSTs is to catalyze toxic electrophilic compounds to react readily with endogenous GSH, thereby protecting other electrophilic centers (e.g., DNA and proteins, etc.) from being damaged (Hayes and McLellan, 1999). Previous studies suggested that *GSTs* could serve as sensor for oxidative stress in response to environmental contaminants including metals (Lee et al., 2017a). In this study, after 30 days of MeHg exposure, the enhanced expression of *GST3* indicated the response of cells to oxidative stress and this result is also matched with the increased GSH content. Similarly, most of the mRNA levels of antioxidant-related genes, including *GSTs*,were also up-regulated by MeHg exposure in a concentration-dependent manner in *Brachionus koreanus* (Lee et al., 2017a). Furthermore, in Atlantic cod (*Gadus morhua*), *GSTs* are involved in consistently up-regulated genes induced by MeHg which suggested the increasing synthesis of antioxidants (Yadetie et al., 2013). Thus, mRNA expression level of *GST3* also have the potential to be used as a sensitive biomarker for MeHg exposure.

The *Hsp70* proteins are critical for protein folding and protecting cells from stress, its production is correlated with thermal stress like high temperatures and metals including cadmium, copper, mercury, etc. (Rajeshkumar and Munuswamy, 2011). Studies found that certain *Hsp* genes including *Hsp70* can act as biomakers by contaminants exposure (Yadetie et al., 2013). As a pleiotropic inhibitor of apoptotic cell death, the protective effect of *HSP27* is mainly in stressed cells (Bruey et al., 2000). *Hsp27* can enhance the antioxidant activities by increasing GSH content. Moreover, it could activate protein kinase B/Akt, and Akt was cell-death inhibitor which can phosphorylate and inactivate the procaspase-9 or prevent the release of cytochrome C from mitochondria, thus reducing the cell death (Mehlen et al., 1996; Konishi et al., 1997; Kennedy et al., 1999). In the present study, the mRNA expression levels of *Hsp70*, *Hsp27* was significantly up-regulated after treatment of MeHg. Similarly, up-regulation of *HSP70* was also observed in liver of Atlantic cod (*Gadus morhua*) suggesting the adaptive responses to MeHg toxicity (Yadetie et al., 2013).

## Conclusion

It changes in enzymes activity and mRNA expressions related to immune response and oxidative stress indicated that MeHg can induce oxidative stress in tissues of large yellow croaker juveniles. Moreover, decreased AChE activity shows MeHg may also induce neurotoxicity effects to fish. On the other hand, the results also suggested that large yellow croaker juveniles have the inherent potential to regulate the levels of antioxidants such as SOD, CAT, GSH, etc. and initiate immune response in order to protect fish to some extent from oxidative stress induced by MeHg. Toxic effects under sub-lethal concentration of contaminants often reflect the ecological, physiological, biochemical, histopathological, or behavioral changes of biota. These responses tend to be latent, but may reduce the ability of organisms to survive, reproduce and compete in natural environment. Since these reactions are not easy to detect, the effects on biological populations are severe. In the future study, we will pay more attention to the behavior and histopathology change of fish tissues thus to know more about how fish juveniles responded to heavy metals. Moreover, this research could offer important information to the development of water quality standards in habitat of large yellow croaker in China.

## Author Contributions

WH designed the experiment. WH and FW analyzed the experimental results and wrote the manuscript. XiX, QL, XuX, and JZ helped in correcting the first and final manuscript. LC and JH helped in writing and revising the final manuscript. YG and SJ helped in keeping the juveniles’ samples and recorded the results of the experiment. All authors have given approval to the final version of the revised manuscript.

## Conflict of Interest Statement

The authors declare that the research was conducted in the absence of any commercial or financial relationships that could be construed as a potential conflict of interest.
